# Should dolutegravir always be withheld in people with HIV on dolutegravir with incident diabetes mellitus? A case report

**DOI:** 10.21203/rs.3.rs-3218404/v1

**Published:** 2023-08-25

**Authors:** Frank Mulindwa, Barbara Castelnuovo, Nele Brusselaers, Robert Bollinger, George Yendewa, Willington Amutuhaire, Claudine Mukashaka, Jean-Marc Schwarz

**Affiliations:** Makerere University Infectious Diseases Institute; Makerere University Infectious Diseases Institute; Antwerp University; Johns Hopkins University; Case Western Reserve University; Case Western Reserve University; Makerere University Infectious Diseases Institute; University of California San Francisco

**Keywords:** HIV, Integrase Inhibitors, dolutegravir, accelerated hyperglycemia, Type 2 Diabetes Mellitus

## Abstract

Dolutegravir (DTG), an integrase strand transfer inhibitor is currently the recommended first and second line anti-retroviral therapy (ART) anchor agent by the World Health Organization. This followed widespread reports of primary resistance to non-nucleoside reverse transcriptase inhibitors.

Despite its very good side effect profile, there have been multiple case reports of ART experienced patients developing hyperglycemia within weeks to a few months after switching to DTG preceded by weight loss. At population level, however, dolutegravir as well as other integrase inhibitors have been demonstrated to have a reduced risk of incident diabetes mellitus (T2DM) compared to other HIV drug classes.

Following multiple similar reports of accelerated hyperglycemia in Uganda during the first pilot year of DTG use, the Uganda Ministry of Health recommended withholding dolutegravir in all patients who develop diabetes. Whether this recommendation should be applied to all patients with incident T2DM remains to be demonstrated.

We present a clinical case of an HIV positive ART naïve man who was diagnosed with T2DM after 36 weeks on dolutegravir. We describe changes in blood glucose, glycated hemoglobin, insulin resistance and pancreatic beta cell function before and after withholding DTG. We demonstrated that he was phenotypically different from the reported cases of accelerated hyperglycemia and he continued to have worsening insulin resistance despite withholding DTG. His blood glucose improved with dietary T2DM management. It is possible he had an inherent risk of developing T2DM independent of his exposure to DTG. This put in question whether DTG should universally be withheld in PLHIV with incident T2DM in Uganda.

## Background

Integrase strand transfer inhibitors (INSTIs) have been demonstrated to have reduced risk of incident type 2 diabetes mellitus (T2DM) compared to non-nucleoside reverse transcriptase inhibitors (NNRTIs) and protease inhibitors (PIs) in different sub-populations in two meta-analyses[1], [2]. The risk was heightened in African populations which were generally under-represented[2] and multiple case reports of patients presenting with accelerated hyperglycemia within weeks to a few months of starting INSTIs have been published[3]–[7]. Following these case reports, the Uganda Ministry of Health guidelines recommended dolutegravir substitution in all patients with incident diabetes mellitus (T2DM)[8]. In this case report, we describe changes in blood glucose, glycated hemoglobin, insulin resistance and pancreatic beta cell function before and after withholding DTG in a patient who was diagnosed with T2DM at 36 weeks on DTG.

## Case presentation

This 44-year-old man was enrolled in a prospective cohort study (**GL**ucose **Me**tabolism changes in Ugandan HIV patients on **D**olutegravir, **GLUMED** study) evaluating glucose metabolism changes in antiretroviral therapy (ART) naïve Ugandan people with HIV (PWH) on tenofovir/lamivudine/dolutegravir (TDF/3TC/ DTG) for 48 weeks[9]. Patients underwent serial 2-hour oral glucose tolerance tests (OGTTs) at baseline, 12 and 36 weeks.

At enrollment on ART, he reported no other known chronic illnesses or long-term medication. He was under-weight with a body mass index (BMI) of 17.5kg/m^2^, had a CD4 + cell count of 261cells/mm^3^, normal creatinine of 0.68 mg/dl, fasting blood glucose (FBG)-88.2 mg/dl and 2-hour blood glucose (2hBG) of 153 mg/dl. He had negative anti-glutamic acid decarboxylase (anti-GAD) and positive anti-Islet cell antigen 2 (anti-IA2) antibody tests. His baseline insulin resistance and pancreatic beta cell function calculated by homeostatic modelling were: HOMA IR −0.6 (normal < 2) and HOMA% β−68% (normal = 100%) respectively[10], [11].

**Figure 1:**
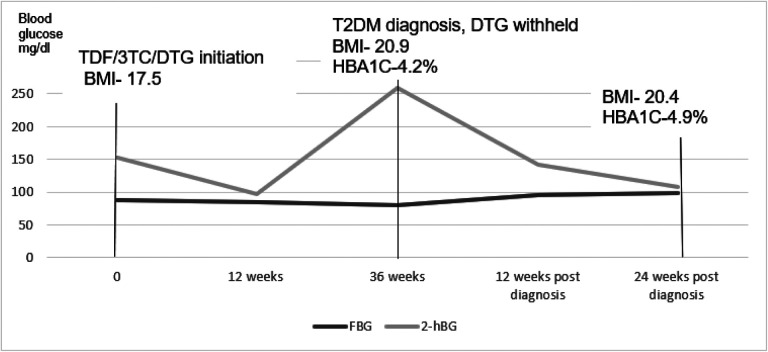
Changes in fasting and 2-hour blood glucose before and after withholding dolutegravir TDF/3TC/DTG-tenofovir/lamivudine/dolutegravir, BMI-Body Mass Index, T2DM-Type 2 Diabetes Mellitus, HBA1C-Glycated hemoglobin

**Figure 2:**
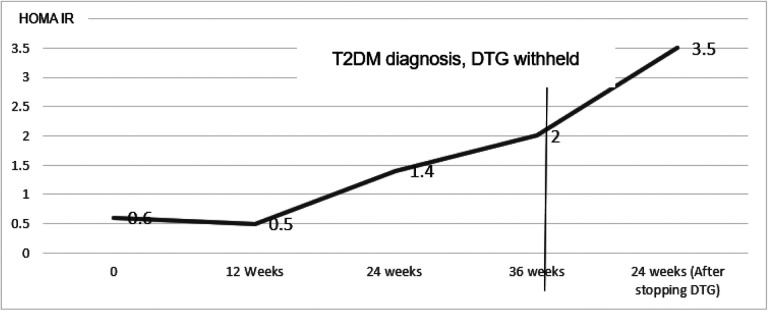
Changes in insulin resistance calculated by Homeostatic modelling (HOMA IR) before and after withholding dolutegravir HOMA IR-Homeostatic model of insulin resistance, DTG-Dolutegravir, T2DM-Type 2 Diabetes Mellitus

**Figure 3:**
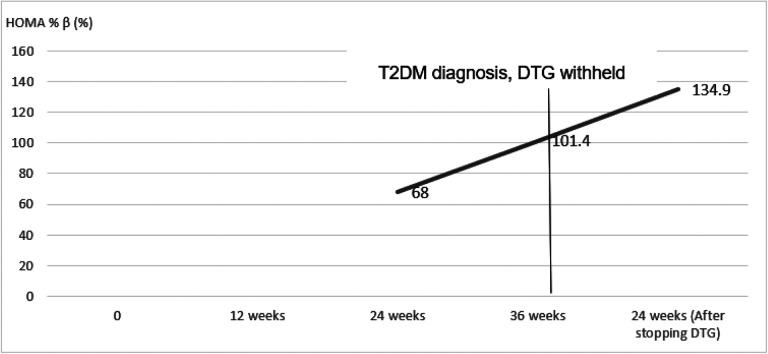
Changes in pancreatic beta cell function calculated by Homeostatic modelling (HOMA %β) before and after with-holding dolutegravir HOMA %β-Homeostatic model of pancreatic beta cell function, DTG-Dolutegravir, T2DM-Type 2 Diabetes Mellitus. (HOMA %β at baseline and 12 weeks was not calculated because the serum insulin was less than the HOMA calculator threshold)

At 36 weeks, on routine evaluation (no symptoms of hyperglycemia) he was diagnosed with T2DM basing on a 2hBG of 259 mg/dl. His BMI had increased to 20.9. Other tests included: HBA1C-4.2%, HOMA IR-2, HOMA% β−101.4%. DTG was substituted with efavirenz according to the Uganda HIV treatment guidelines[8]. He was managed on diet adjustment without T2DM pharmacologic intervention. Serial clinical and blood glucose evaluations at 2,4, 8, 12 and 24-weeks post T2DM diagnosis were normal. At 24 weeks, his HBA1C had increased to 4.9% much as blood glucose had normalized (FBG-99mg/dl and 2hBG-108mg/dl). Insulin resistance continued to worsen with a HOMA IR of 3.5% while his pancreatic beta cell function continued to increase with HOMA %β = 134.9%. Dietary non pharmacological management was continued after 24 weeks. Changes in BMI, blood glucose, HBA1C, HOMA IR and HOMA % β have been summarized in Figs. 1,2 and 3.

## Discussion and conclusions

Review of current literature suggests that at population level, integrase inhibitors are associated with a reduced risk of incident diabetes mellitus compared to NNRTIs and PIs[1], [2]. There is also evidence to demonstrate insignificant differences in effects on insulin resistance compared to other HIV drug classes[2]. Additionally, there are published case reports documenting that a certain section of heavily ART experienced PWH, probably with a currently unclear predisposition develop accelerated hyperglycemia when exposed to integrase inhibitors[3]–[7]. These patients typically present with diabetic ketoacidosis preceded by weight loss, a phenotype typical of insulin deficiency states, but with normal C-peptide levels as summarized in Table 1. In these patients, the temporality between introduction of INSTIs and development of hyperglycemia is easily demonstrable. Similarly, withholding INSTIs has been demonstrated to lead to markedly reduced T2DM pharmacological treatment requirements and complete resolution of hyperglycemia in some cases (Table 1).

The patient we present had an asymptomatic onset of glucose intolerance over 36 weeks (diagnosed on routine screening) and gained weight before diabetes diagnosis in contrast to the above group of patients. Various factors such as immune reconstitution, improved appetite as well as dolutegravir itself could have contributed to the weight gain[12], [13]. Over the same period, he experienced worsening insulin resistance and concurrent increase in pancreatic beta cell function which could have been compensatory. Much as the worsening insulin resistance could be attributed to the weight gain, on evaluation of glucose changes in the whole study cohort, there was significant improvement in blood glucose despite a significant increase in BMI over the 48 weeks of follow up[9]. This may suggest that this patient had an inherent risk of developing T2DM, independent of weight gain and exposure to DTG. Blood glucose after T2DM diagnosis may have improved because of the dietary modification as well as compensatory hyperinsulinemia. It can as well be argued that blood glucose may have improved because DTG was discontinued but this may be unlikely because of the continued worsening insulin resistance and probable compensatory hyperinsulinemia.

In conclusion, this case report suggests that dolutegravir should not be universally withheld in ART naïve PWH who develop diabetes mellitus but the decision made on a case-by-case basis. More research is however needed to ascertain these findings. This is programmatically pertinent in sub-Saharan Africa given dolutegravir is first line treatment in majority HIV programs with millions of PWH on DTG due to wide spread primary resistance to NNRTIs[14].

## Figures and Tables

**Table 1:** Summary of published case reports of accelerated severe hyperglycemia in people with HIV on integrase inhibitors

Author (Country of origin of patients)	Baseline patient demographic and clinical characteristics	Clinical presentation	INSTI withheld?
Nathanial et al[5] (North America)	25 y/male, ART experienced, switched to BIC/TAF/FTC HBA1 C-6.6%, FBG-85mg/dl	After 4 months DKA HBA1C->17% Normal C-peptide	Yes Marked improvement in glycemic control after withdrawal
	56y/male, ART experienced, switched to BIC/TAF/FTC HBA1 C-6%	After 2months: DKA HBA1 C-12.6% Normal C-peptide preceding weight loss of 15kg	Yes Marked improvement in glycemic control after withdrawal
	41y/male, ART experienced, switched to BIC/TAF/FTC FBG-78mg/dl HBA1C-5.6%	After 2 months: DKA Normal C-peptide HBA1C-12.4%	Yes Marked improvement in glycemic control after withdrawal
Lamorde et al[4] (Uganda)	16 patients, 15 ART experienced, 1 ART naïve Initiated on Dolutegravir anchored ART Baseline blood glucose, HBA1C not measured	Mean duration beforepresentation to the ER: 4 months DKA preceding weight loss	Yes Marked improvement in glycemic control after withdrawal
Fong et al[7] (North America)	44y/male ART experienced Switched to RAL HBA1C-2 months prior to switch - 5.5%	After 4 months: DKA HBA1 C-9.3% Normal C-peptide preceding weight loss	Yes Marked improvement in glycemic control after withdrawal
McLaughlin et al[6] (North America)	ART experienced Switched to DTG Known to have stage 4 CKD, Type II Diabetes Mellitus, Hypertension HBA1C-6.2%, FBG-65mg/dl	After 3 weeks: Hyperosmolar Hyperglycemic state, RBG-949mg/dl, HBA1C-14.9%.	Yes Marked improvement in glycemic control after withdrawal.
Kamal et al[3] (North America)	ART experienced, defaulted in 2013. started on TDF/FTC/DTG in June- 2018 HBA1 C-5.9%	After 1 month: On routine evaluation- RBG = 467mg/dl (started on metformin 500mg) After 2nd month: Admitted with Hyperosmolar Hyperglycemic state, HBA1C- 12.9%, started on insulin.	Not mentioned.

y-years ART-Anti-retroviral Therapy, BIC-bictegravir, TAF-tenofovir alafenamide fumarate, FTC-Lamivudine, DTG-dolutegravir. FBG-fasting blood glucose, HBA1C-glycated hemoglobin, DKA-diabetic ketoacidosis, RAL-raltegravir, RBG-random blood glucose, CKD-chronic kidney disease.

## Data Availability

The datasets used and/or analyzed during the current study are available from the corresponding author on reasonable request.

## Abbreviations

DTG: dolutegravir
T2DM: Type 2 Diabetes Mellitus
ART: Anti-retroviral therapy
INSTIs: Integrase strand transfer inhibitors
NNRTIs: Non-nucleoside transcriptase inhibitors
PIs: Protease inhibitors
CD4: Cluster of differentiation 4
HOMA IR: Homeostatic model for insulin resistance
HOMA %β: Homeostatic model for pancreatic beta cell function
FBG: Fasting blood glucose
2hBG: 2-hour blood glucose
HBA1C: Glycated hemoglobin
